# Development of a Highly Sensitive and Specific Immunoassay for Determining Chrysoidine, A Banned Dye, in Soybean Milk Film

**DOI:** 10.3390/molecules16087043

**Published:** 2011-08-17

**Authors:** Hongtao Lei, Jin Liu, Lijun Song, Yudong Shen, Simon A. Haughey, Haoxian Guo, Jinyi Yang, Zhenlin Xu, Yueming Jiang, Yuanming Sun

**Affiliations:** 1 Guangdong Provincial Key Laboratory of Food Quality and Safety, South China Agricultural University, Guangzhou 510642, Guangdong, China; Email: hongtao@scau.edu.cn (H.L.); liujin_001163@163.com (J.L.); songlijun0703330@163.com (L.S.); shenyudong@scau.edu.cn (Y.S.); ghxghz@sina.com (H.G.); yjy361@163.com (J.Y.); jallent@163.com (Z.X.); 2 Institute of Agri-Food and Land Use, Queen’s University Belfast, Belfast BT9 5AG, Northern Ireland, UK; Email: s.a.haughey@qub.ac.uk (S.A.H.); 3 Key Laboratory of Plant Resources Conservation and Sustainable Utilization, South China Botanical Garden, Chinese Academy of Sciences, Guangzhou 510650, Guangdong, China

**Keywords:** chrysoidine, antibody, ELISA, soybean milk film

## Abstract

A highly specific and sensitive indirect competitive enzyme-linked immunosorbent assay (icELISA）was developed for the first time for the detection of chrysoidine, a dye banned in soybean milk film. Two haptens with different spacer arms were synthesized to produce antibodies. Both homologous and heterologous immunoassay formats were compared to enhance the icELISA sensitivity. The heterologous icELISA exhibited better performance, with an IC_50_ (50% inhibitory concentration) of 0.33 ng/mL, a limit of detection (LOD, 10% inhibitory concentration) of 0.04 ng/mL, and a limit of quantitation (LOQ, 20%–80% inhibitory concentration) from 0.09 to 4.9 ng/mL. The developed icELISA was high sensitive and specific, and was applied to determine chrysoidine in fortified soybean milk film samples. The results were in good agreement with that obtained by high-performance liquid chromatography (HPLC) analyses.

## 1. Introduction

**Figure 1:**
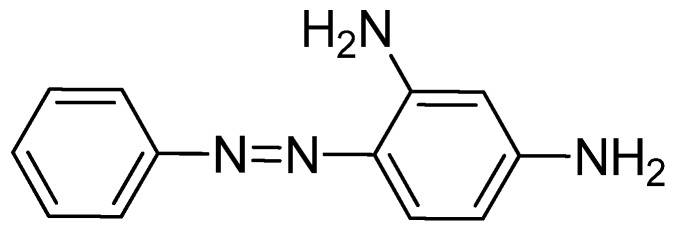
Structure of chrysoidine.

Chrysoidine (C.I. Basic Orange 2, Figure 1) is a type of industrial azoic dye [1]. Due to its good dyeing fastness, it is widely used for dyeing leather, paper, feather, grass, wood, bamboo, *etc.* [2]. Chrysoidine can cause acute and chronic toxicity to mammals when taken by oral or skin route, or inhaled, and its median lethal concentration (LC_50_, 24 h) for fish was 0.5 mg/L [3]. Chrysoidine has also been recognized as a carcinogen [4] and its use in food has not been approved by any country. Unfortunately, it has been reported that soybean milk film, a popular soybean food consumed in China was adulterated with chrysoidine [5,6]. Moreover, chrysoidine has also been found in yellow-fin tuna and dried bean curd stick [2,7]. Therefore, control of this banned dye in food is very crucial and the development of a simple, economic, and rapid detection method is urgently needed.

The reported analyses of chrysoidine were mainly physio-chemical methods based on chromatography with various detectors [2,4,5,8,9]. The detection limit of chrysoidin in a HPLC-MS study was 0.25 ng/g [4], and 2.3 ng/g in a GC-MS method [8]. Chromatography methods can provide accurate and reliable results, but these methods are also expensive, laborious and time-consuming [10]. To date, immunoassay technologies, especially ELISA, are increasingly replacing traditional chemical analyses in screening of food contaminants and agrochemicals due to their sensitivity, time-efficiency and cost-effectiveness [11,12,13]. ELISAs have been widely used for the determination of various contaminants such as toxins [14,15], drugs [16,17], pesticides [18] and illegal additives [11,12] in the biological, agriculture, and environmental fields. However, to the best of our knowledge, there is no published literature on an immunoassay for the detection of chrysoidine.

In this study, a highly sensitive and specific ELISA for the determination of chrysoidine was developed for the first time. Two chrysoidine haptens with different spacer arm lengths were synthesized and covalently coupled to different carrier proteins to produce both immunogens and coating antigens. The polyclonal antibody (pAb) to chrysoidine raised from immunized rabbits were characterized and used for a competitive ELISA. The developed ELISA was further employed to analyze spiked soybean milk film samples and validated by a HPLC method.

## 2. Results and Discussion

### 2.1. Synthesis of Chrysoidine Haptens

**Scheme 1:**
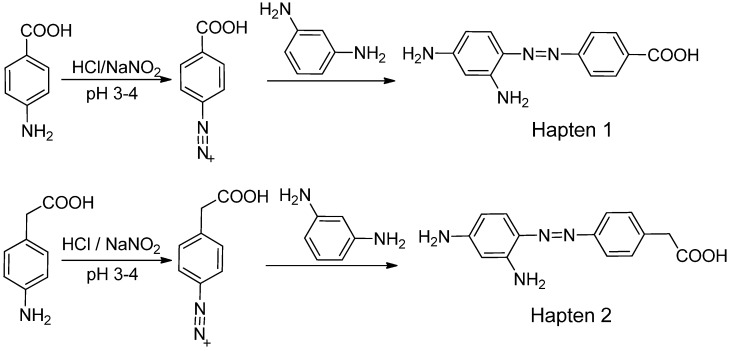
Synthesis of chrysoidine haptens.

For production of high quality antibodies and development of highly sensitive and specific immunoassays, it is important to design a proper hapten structure. It was proposed that both the conjugation position at the hapten molecule where the spacer is attached and the length of the spacer may play an important role for a successful antibody production [11,19], and that the molecular structure of the hapten should be left unchanged [20]. In this study two chrysoidine-derivatives with different spacer lengths were synthesized (Scheme 1). One derivative with one carbon-atom spacer length (Hapten 1) and the other with two-carbon-atom spacer length (Hapten 2) were modified at the *para* position of the azo bond. The structures of Hapten 1 and Hapten 2 were confirmed by thin layer chromatography (TLC), mass spectrometry (MS) and nuclear magnetic resonance (NMR) methods.

### 2.2. Synthesis of Immunogen and Coating Antigen

**Figure 2:**
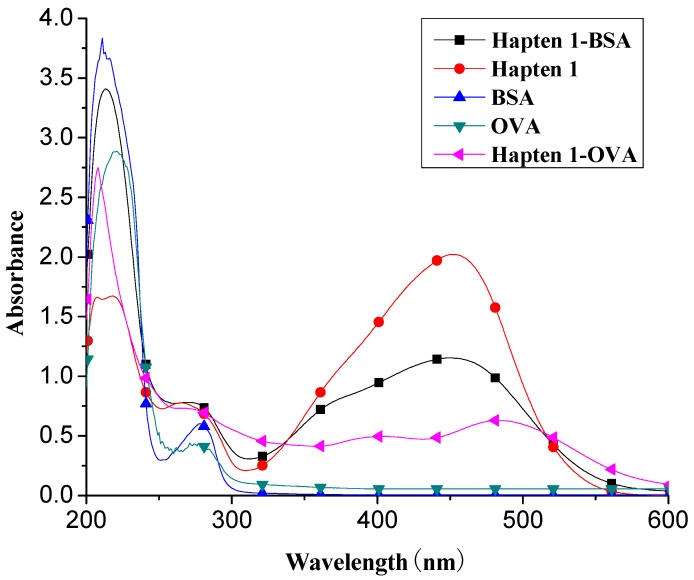
UV spectra of BSA, OVA, Hapten 1, Hapten 1-BSA and Hapten 1-OVA.

The chrysoidine derivative bearing a carboxylic acid group at the end of the spacer was activated by the active ester method and then covalently coupled with a carrier protein (BSA or OVA) [21]. The conjugates of hapten-BSA and hapten-OVA were used as immunogen and coating antigen, respectively. Figure 2 shows the UV spectra of BSA, OVA, Hapten 1, Hapten 1-BSA and Hapten 1-OVA with absorption peaks of Hapten 1, BSA and OVA at 450, 280 and 280 nm, respectively. The absorption spectra of Hapten 1-BSA/OVA conjugates contain both absorption peaks of Hapten1 and BSA/OVA, but with somewhat red shift. The results indicated that the coupling of hapten to BSA and OVA was successful. Similar results were obtained with Hapten 2 conjugate (data not shown).

### 2.3. Optimization of icELISA Conditions

The prepared coating antigens were used for the icELISA assay. To improve the sensitivity of icELISA, we have optimized the assay conditions by changing the assay format, concentration of the coating antigen, dilution of the antiserum and antigen antibody reaction time. Two criteria were used for optimizing the icELISA assay. (1) obtain a minimum IC_50_ value and (2) increase an absorbance to 0.8–1.5 units for the zero standard concentration (blank) [22].

**Figure 3:**
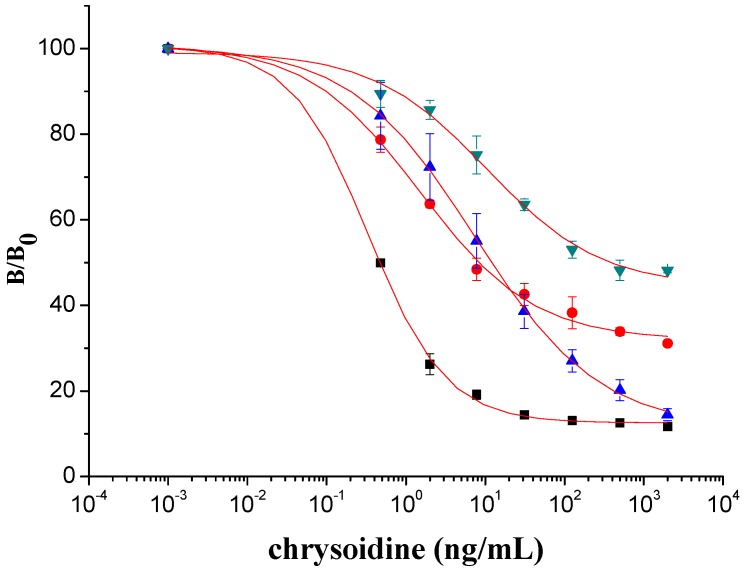
icELISA standard curves for chrysoidine with heterogeneous combinations of coating antigen and antibody. Data represented in mean ± SD (standard deviation) from three assays. ■ combination of antibody 2 and Hapten 1-OVA; ● combination of antibody 1 and Hapten 2-OVA; ▲ combination of antibody 1 and Hapten 1-OVA; ▼ combination of antibody 2 and Hapten 2-OVA.

The antisera collected after each boost were subject to titration and inhibition by the homology indirect and competitive ELISA, respectively. All antisera showed relatively constant high titer and inhibition ratio after the sixth immunization. Therefore, combinations of two different coating antigens (Hapten 1-OVA and Hapten 2-OVA) and two antibodies (Ab 1 from Hapten 1 and Ab 2 from Hapten 2) were tested. The experimental conditions, including coating antigen concentration and antiserum dilution, were optimized for each combination (Table 1). In specific, coating antigen concentration and antiserum varied in the range of 17–20 ng/mL and 1:4,000–1:20,000, respectively. The calculated IC_50_ values were found to be less than 11 ng/mL. With an IC_50_ of 0.33 ng/mL, the combination of hapten 1-OVA and Ab 2 was found to be most sensitive. With this combination the typical standard curve for chrysoidine were constructed in the concentration range from 0.09 to 4.9 ng/mL and the values of LOD at 10% inhibition was within 0.04 ng/mL, indicating that the sensitivity was better than that of the reported physio-chemical method [4]. In addition, since both heterologous combination (Ab 1/hpaten 2-OVA and Ab 2/hapten 1-OVA) demonstrated lower IC_50_, significant differences in assay sensitivity were observed between homogeneous and heterogeneous combinations of coating antigens and antibodies (Figure 3), which implied that the heterologous plate coating antigens significantly enhanced the assay sensitivity [12]. Thus, the combination of Ab 2 and hapten 1-OVA was used for the further investigation due to its better sensitivity.

**Table 1:** Optimized icELISA conditions.

Coating antigen name	Coating concentration (ng/mL)	Antibody name	Antibody dilution	IC_50_ (ng/mL)	LOD (ng/mL)
Hapten 1-OVA	20	Ab 1	8000	7.4	0.8
Hapten 1-OVA	20	Ab 2	4000	0.33	0.04
Hapten 2-OVA	20	Ab 1	20000	1.6	0.4
Hapten 2-OVA	17	Ab 2	10000	10.6	3.8

### 2.4. Specificity of the icELISA

The assay specificity was evaluated using a set of compounds structurally related to chrysoidine, and the cross-reactivity data for each compound is given in Table 2. The highest recognition was raised from the two artificial haptens with CR values of 170%, 127%, respectively. The high CR to the haptens could be explained by the similarity of the molecular structure [13]. The icELISA showed lower cross-reactivity to Acid Yellow 23, orangeim and auramine O, the CR values were 0.04%, 0.12% and 0.11%, respectively, which may be associated with the shared group benzene-ring, azo-bond and amino group among these compounds. From Table 2 it is also apparent that there was no cross-reactivity with other compounds, where CR values were less than 0.01%. As expected, the major difference in the structures between chrysoidine and other dyes should be responsible for the low or no recognition [11,23,24,25]. Although Hapten 1 and 2 showed more than 100% cross-reactivity, they both are artificial and, therefore, have seldom chance to occur in food to cause the matrix effects. As a result, the developed icELISA can meet the specificity requirement for the screening assay of chrysoidine.

**Table 2:** Cross-reactivity of the polyclonal Ab 2 with chrysoidine, hapten and other compounds based on coating antigen Hapten 1-OVA.

Compounds	Structure	IC_50_ (nmol/L)	Cross-reactivity (%)
Chrysoidine	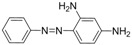	1.33	100.0
Hapten1	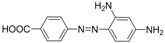	0.78	169.8
Hapten2	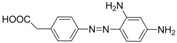	1.05	127.0
Sunset yellow	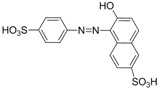	^a^ ND	≤0.01
Acid Yellow 23	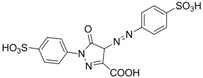	3054.4	0.04
Orangeim	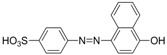	1121.90	0.1
Acid Red 94	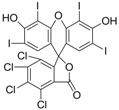	ND	≤0.01
Auramine O	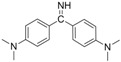	797.81	0.2
1-(4-nitrophenyl-azo)-2-naphthol	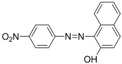	ND	≤0.01
Sudan Red I	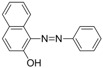	ND	≤0.01
Azorubine	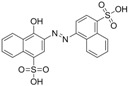	ND	≤0.01
Indigo	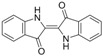	ND	≤0.01
Allura Red	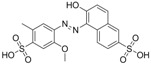	ND	≤0.01
Quinoline yellow	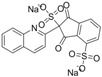	ND	≤0.01
Erythrosine	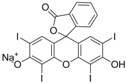	ND	≤0.01
Tartrazine	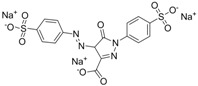	ND	≤0.01
Amaranth	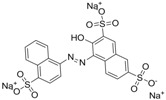	ND	≤0.01
Carminic acid	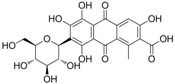	ND	≤0.01
Scarlet Base G	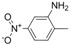	ND	≤0.01
Reddish orange	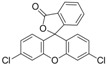	ND	≤0.01
Carmine	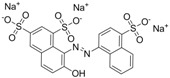	ND	≤0.01
Brilliant blue-85	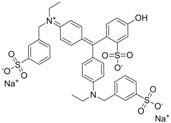	ND	≤0.01
Orange yellow	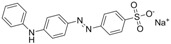	ND	≤0.01
Gardenia yellow	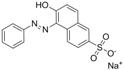	ND	≤0.01
Melamine	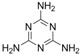	ND	≤0.01

^a^ ND represents the infinite IC_50_ value that could not be fitted with the four-parameter logistic equation.

### 2.5. Fortification Experiment

The performance of an immunochemical method can be significantly influenced by the various complicated matrices existing in the sample, and a dilution is usually a good way to eliminate this matrix effect [26]. The effects caused by matrix were investigated using soya milk film purchased from a local market (confirmed to be negative by HPLC). In this study, soybean milk film samples were fortified with chrysoidine at a concentration of 45–180 ng/g and extracted with the extraction buffer (0.1 M Na_2_HPO_4_:MeOH = 60:90, v:v). The absorbance of the 60-fold diluted sample extraction was found to be almost same to that of the PBST (data not shown), indicating no obvious matrix effect. Accordingly, the 60-fold dilution was selected for the subsequent experiments. The results obtained from each spiked sample with five replicates are shown in Table 3. Recovery rates of 102.1%–106.5% and intra-assay coefficients of variation of 2.4–4.9% were obtained.

**Table 3:** Recovery of chrysoidine from the fortified samples by icELISA.

Sample weight (g)	Chrysoidine added (µg)	Fortified con. (ng/g)	Dilution of extract	Detected (ng/g)	Recovery (%)	CV (%) (*n =* 5)
2.5	0.112	45	60	48	106.5	4.9
2.5	0.225	90	60	92	102.1	3.4
2.5	0.450	180	60	186	103.1	2.4

### 2.6. Validation of icELISA by HPLC

**Figure 4:**
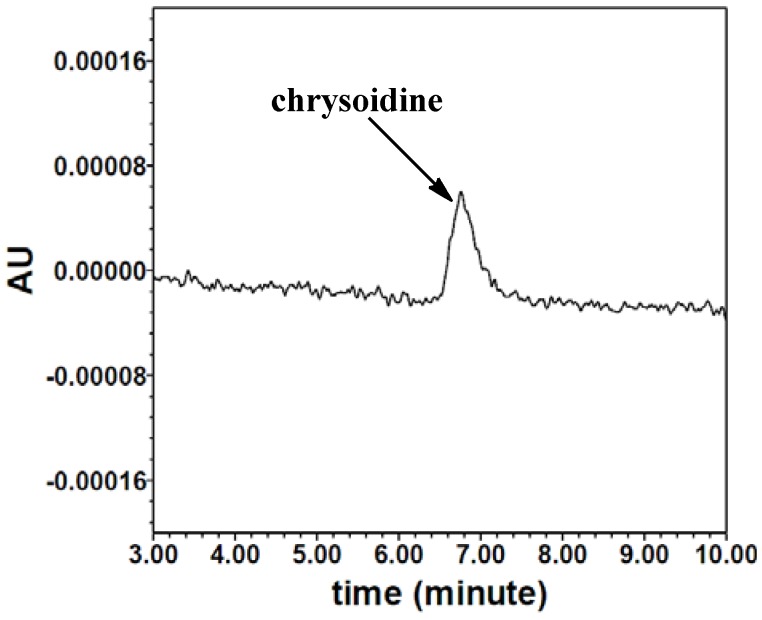
HPLC chromatogram of chrysoidine (10 ng/mL).

The HPLC calibration curve for chrysoidine was constructed in the range of 0, 10, 20, 30, 90, 270, 810 and 2430 ng/mL. As indicated in Figure 4, chrysoidine can be detected at the retention time of 7.0 min using HPLC. The linear equation of the HPLC standard curve was Y = 122.4X − 359.0 (*r^2^* = 0.99). The detection and quantification limits, which were respectively defined as signal-to-noise ratio of 3:1 and 10:1, were 6.4 and 14.6 ng/mL, respectively.

**Figure 5:**
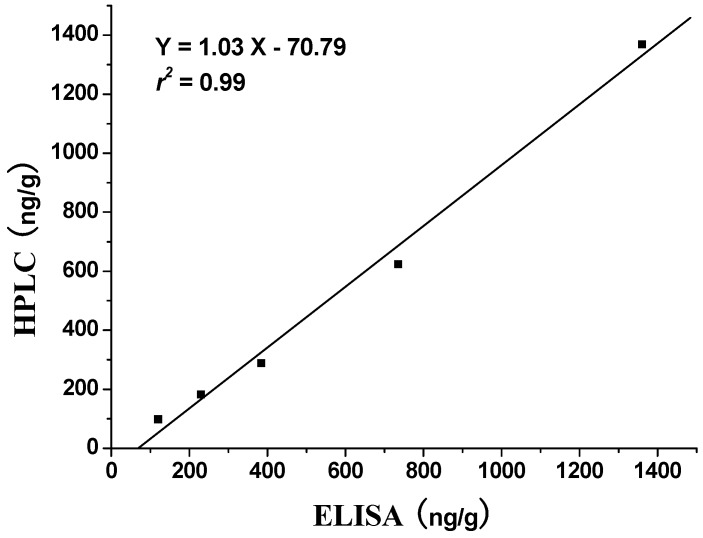
Correlation of icELISA with HPLC analysis for soybean milk film samples.

To validate the applicability of the icELISA, the fortified food samples were measured by HPLC and icELISA simultaneously. As shown in Figure 5, there is a good correlation between icELISA and HPLC with the linear regression equation of Y = 1.03X – 70.79 (*r^2^* = 0.99, *n* = 5). These results show that icELISA can be useful for the primary quantitative screening of chrysoidine in soybean milk film.

## 3. Experimental

### 3.1. Reagents

General reagents and organic solvents were of analytical grade unless specified. Chrysoidine (HPLC grade), *N,N’*-dicyclohexylcarbodiimide (DCC), *N*-hydroxysuccinimide (NHS), 3,3’,5,5’-tetramethyl-benzidine (TMB), bovine serum albumin (BSA), ovalbumin (OVA), 4-aminobenzoic acid, 4-aminophenylacetic acid, 3-aminobenzoic acid, *m-*phenylenediamine, complete and incomplete Freund’s adjuvants were purchased from Sigma (St. Louis, MO, USA). HRP-conjugated goat-anti-rabbit IgG was obtained from Boster Biotech Corporation Limited. (Wuhan, China). Polystyrene ELISA plates were provided from Jincanhua Corporation (Shenzhen, China). Tween-20, methanol (HPLC grade), dimethylformamide (DMF), dimethyl sulfoxide (DMSO) were obtained from Yunhui Company (Guangzhou, China). All structure-related compounds such as Sunset yellow, Acid Yellow 23, Orangeim, Acid Red 94, Auramine O, 1-(4-nitrophenylazo)-2-naphtho and Sudan Red I, *etc.*, used for the cross-reactivity evaluation, were purchased from Dongzheng Chemical Corporation (Guangzhou, China). Coating buffer: 0.05 M carbonate buffer, pH 9.8; Assay and washing buffer (PBST): 0.01 M phosphate buffered saline with Tween-20, pH 7.4, containing 145 mM NaCl and 0.05% (v:v) Tween-20; Blocking solution: 5% (v:v) skim milk powder in distilled water; Substrate solution: 15 mg TMB in 10 mL DMF, keep in dark place at 4 °C; Developing buffer: 0.04 M Na_2_HPO_4_·12H_2_O and 0.048 M citric acid; Stop solution: sulfuric acid (10%, v/v); Extraction buffer: 30 mL 0.1 M Na_2_HPO_4_, 150 mL H_2_O and 270 mL methanol to form 450 mL extraction buffer.

### 3.2. Instrumentation

UV-3010 spectrophotometer (Hitachi, Japan) was used for the UV-visible absorption measurements. ELISA plates were washed with a microtiter plate washer DEM-3 (Tuopu, China). Absorbance was measured at a wavelength of 450 nm using the Wallac 1420 VICTOR3 multilabel counter (Perkin Elmer Ltd., US).

### 3.3. Hapten Synthesis

Two haptens used for immunizing and coating antigen are shown in Scheme 1. They were synthesized by the same procedure using corresponding commercial start materials. The following subsections describe the synthesis procedure and the identification of haptens.

#### 3.3.1. *4-((2,4-Diaminophenyl)diazenyl)benzoic acid* (Hapten 1)

To a stirring solution of 4-aminobenzoic acid (0.411 g, 3 mmol) in water (10 mL), cooled in an ice water bath, 0.1 M hydrochloric acid (10 mL) was added to keep pH at 3–4, followed by dropwise addition of sodium nitrite (0.228 g) dissolved in water (1 mL). The mixture was stirred for 2 h, and then *m-*phenylenediamine (0.356 g, 3.3 mmol) dissolved in water (5 mL), pH adjusted to 6–7 with hydro-chloric acid, and cooled with ice water was added dropwise. After stirring for 2 h, the mixture was kept at 4 °C overnight and filtered to collect the residue. The crude product was purified by silica gel column chromatography (chloroform-methanol = 15:1, v:v). Yield: 62%. TLC *R_f_* =0.5 (CHCl_3_:MeOH = 15:1); MS (APCI positive) *m/z*: 257 [M+H]^+^. ^1^H-NMR (DMSO-*d_6_*, 600 MHz) δ: 8.79 (s, 1H), 8.58 (s, 1H), 7.95 (d, 2H), 7.82 (d, 4H), 6.50 (d, 4H), 3.59 (d, 2H).

#### 3.3.2. *2-(4-((2,4-Diaminophenyl)diazenyl)phenyl)acetic acid* (Hapten 2)

Hapten 2 was synthesized using 2-(4-aminophenyl)acetic acid as the starting compound (Scheme 1). The procedure is the same as the synthesis of Hapten 1 above. TLC *R_f_* = 0.5 (CHCl_3_:MeOH = 15:1). MS (APCI positive) *m/z*: 271 [M+H]^+^. ^1^H-NMR (DMSO-*d_6_*, 600 MHz) δ: 7.61 (d, 2H), 7.29–7.36 (d, 4H), 5.86–6.00 (d, 2H)

### 3.4. Preparation of Immunogen and Coating Antigen

The hapten was conjugated to BSA (bovine serum albumin; for use as immunogen) and OVA (ovalbumin; for use as coating antigen) by the active ester method [12]. Briefly, to the each hapten (0.1 mmol) in DMF (500 µL), DCC (0.2 mmol), followed by NHS (0.2 mmol) was added slowly. This activation reaction was carried out overnight at 4 °C with continuous stirring. The reaction mixture was centrifuged (10,000 g, 10 min) and, the supernatant was added very slowly to a BSA solution (150 mg BSA or ovalbumin dissolved in 15 mL PBS). The mixture was stirred overnight at 4 °C to complete the conjugation reaction. The mixture was then dialyzed against phosphate buffer (0.01 mol/L, pH 7.4) for 3 days (three changes of buffer per day). The immunogen and coating antigen were characterized by the ultraviolet spectrophotometers [13]. The obtained immunogen hapten-BSA or coating antigen hapten-OVA were stored at −20 °C until use.

### 3.5. Immunization Protocol

The intramuscular injection was given to one New Zealand white rabbits for each immunogen, total two rabbits were used to raise antibody. In brief, each immunogen hapten-BSA (0.5 mg) was dissolved in saline and emulsified with an equal volume of Freund’s complete adjuvant. Two New Zealand rabbits weighing 1.5–2.0 kg were immunized four times using emulsified Hapten 1-BSA, Hapten 2-BSA, respectively, at intervals of 21 days by the Guangdong Medical Laboratory Animal Center. The rabbits were blood sampled to detect the presence of antibodies to chrysoidine using an indirect ELISA on the eighth day after each immunization, starting 40 days after the first injection. Bleeding of animals was performed 10 days after the fouth immunization and the collected antisera, named Ab 1 for that raised from Hapten 1-OVA, Ab 2 for that raised from Hapten 2-OVA, respectively. Antisera were divided into aliquots (1 mL) and stored at −20 °C until use.

### 3.6. Indirect Competitive ELISA

Checkerboard assays, in which antibodies were titrated against various amounts of the coating antigen, were used to measure the reactivity of antibodies and to select an appropriate antigen coating and antibody dilutions for competitive indirect assays. From the results of the checkerboard assays, two antibodies were selected as the most suitable ones. Then, to select the most suitable coating antigen, competitive assays were performed under various combinations at several chrysoidine concentration levels. The concentrations of the antibodies and the coating antigen chosen were further optimized.

The procedure for the competitive assay was as follows. Microtiter plates were coated with hapten-OVA (100–1000 ng/mL, 100 µL/well) in carbonate-bicarbonate buffer (50 mM, pH 9.6) by overnight incubation at 4 °C. The plates were blocked with blocking solution by incubation for 3 h. Serial dilution of the analyte in methanol-PBST (1:9, v:v, 50 µL/well) were added, followed by 50 µL/well of antibody diluted (1/1,000–1/10,000) with PBST solution. After incubation for about 30–60 min, 100 µL/well of a diluted (1/10,000) goat anti-rabbit IgG-HRP was added. The mixture was incubated for 60 min, and 100 µL/well of a TMB solution was added. The reaction was stopped after an appropriate time (typically 8 min) by adding 10% (v:v) H2SO4 (50 µL), and the absorbance was read at 450 nm.

### 3.7. Cross-Reactivity

Specificity of the optimized assay was tested by measuring the cross-reactivity using a group of structurally related dyes. Twenty-four compounds were selected for testing the cross-reactivity (CR) (Table 1). The CR was calculated as follows: CR(%) = [IC_50_(chrysoidine) / IC_50_(interferent)] × 100.

### 3.8. Sample Preparation

The soybean milk film samples were obtained from a local food supplier and were homogenized. To the soybean milk film sample (2.5 g) chrysoidine standard (112.5, 225, 450 ng) was added in methanol (100 µL). After setting aside for 1 h, extraction buffer (25 mL) was infused into the sample and the extraction mixture was shaken for 20 min and supersonicated for 20 min, followed by centrifugation (10,000 g) for 10 min. The supernatant was collected and diluted appropriately with PBST to be measured by icELISA. For each sample, three separate extractions were performed and each sample was determined in five replicates. Negative samples confirmed by HPLC were extracted in the same way and used as a blank.

### 3.9. HPLC Analysis

Samples were extracted as described above just without the last PBST dilution step, after a filtration with 0.45 µm membrane chrysoidine was analyzed on a Waters HPLC system equipped with a Waters X-Brige-C18 column (4.6 × 150 mm, 5 µm). The injection volume was 10 µL. The mobile phase consisted of methanol and 5 M ammonium acetate-acetic acid buffer (pH 4.17) at a volume ratio of 55:45. The flow rate was 1.0 mL/min. The detection wavelength was set at 458 nm. The retention time of chrysoidine was 6.7 min. The concentrations of chrysoidine were calculated by calibration with the peak areas of external chrysoidine standard.

## 4. Conclusions

In this work, antibodies against chrysoidine were prepared for the first time and used to develop an indirect competitive ELISA. Two types of hapten have been synthesized and used to produce polyclonal antibodies and coating antigens. Use of the antibody against the immunogen with two-carbon-atom spacer arm and the heterologous coating antigen with one-carbon-atom spacer arm resulted in the high sensitivity and specificity. The IC_50_ and LOD values of the assay for chrysoidine are 0.33 and 0.04 ng/mL, respectively. All tested dyes exhibited no cross-reactivity with the antibody to chrysoidine. In addition, high recovery rates (102.1%–106.5%) were obtained when this assay was applied to the fortified samples. The ELISA method was also compared to HPLC with an excellent correlation. Therefore, the icELISA could be a feasible quantitative/screening method for chrysoidine analysis in food samples.
